# Loosening Monitoring of a Threaded Pipe Connection Using the Electro-Mechanical Impedance Technique—Experimental and Numerical Studies

**DOI:** 10.3390/s18113699

**Published:** 2018-10-30

**Authors:** Yabin Liang, Qian Feng, Dongsheng Li, Sijia Cai

**Affiliations:** 1 Key Laboratory of Earthquake Geodesy, Institute of Seismology, China Earthquake Administration, Wuhan 430071, China; yabinliang@eqhb.gov.cn; 2 Wuhan Institute of Earthquake Engineering, Wuhan 430071, China; jiangjian@eqhb.gov.cn; 3 Department of Civil Engineering, Shantou University, Shantou 515063, China; lids@stu.edu.cn

**Keywords:** threaded connection, loosening monitoring, electro-mechanical impedance (EMI) technique, piezoceramic transducer

## Abstract

Threaded connections are the most common pipe fittings used in oil and gas transportation systems. Due to external vibrations, cyclic loads, and pollution, the fitting parts may start getting loose, which could result in pipeline leaks and other environmental disasters. It is of great significance to develop a reliable technique that could provide real-time monitoring of the looseness of pipeline fittings. In this paper, a piezoceramic-based active sensing method combined with the electro-mechanical impedance (EMI) technique was developed to monitor the health condition of threaded pipe connections in real time. Two pipe segments coupled with a threaded coupling fitting were assembled in the laboratory, and a lead zirconate titanate (PZT) patch was surface bonded onto the coupling part. In the experiment, the PZT impedance signatures were measured at each simulated loosening condition. A root-mean-square deviation (RMSD) method was employed to build a looseness index from the measured impedance signatures. To verify the effectiveness of the developed EMI technique, the experimental results were compared with those computed from a numerical simulation. The good agreement from experimental and numerical results highlights that the developed piezoceramic-based EMI technique has great potential for determining early looseness, as well as for monitoring the health status of the pipeline fitting during its service life.

## 1. Introduction

Threaded connections are commonly used for pipe assemblies as an alternative to welding for those applications in which pipes should be frequently coupled and uncoupled [1], for example, to connect risers, tendons, drill pipes and well casing strings, etc. The connections normally consist of a male and a female part, respectively called the pin and box. In practical applications, this type of connected structure is always subjected to external variable loads and environmental pollution, such as vehicle cyclic loads, which may induce cracks or looseness of the connection during its service period. On the other hand, previous investigations have revealed that failures occurring in threaded connections are the main reason leading to oil tubing and casing accidents [2,3,4]. Therefore, it is very important to ensure the seal quality of pipeline connections so as to reduce the potential leakage risk and guarantee the safety of the entire chain [5]. 

Several techniques have been studied and applied to detect leakages and monitor the health conditions of pipeline structures during their service period. Niklès et al. [6] proposed that the presence of a leak in a pipeline can be detected by a drop in the local pressure measured by pressure sensors. Qu et al. [7] presented a pre-warning system to detect abnormal events of the pipelines by installing distributed optical fiber sensors along the pipeline and monitoring the changes in the vibrational signal. Liang et al. [8] and Zhou et al. [9] developed an optical-fiber-based distributed temperature sensing system to detect and locate leakages along pipelines by monitoring the variation in the pipeline’s surrounding temperatures. On the other hand, for threaded pipe connections, Liang et al. [10] and Hong et al. [11] applied a piezoceramic-based active sensing method combined with the time reversal technique to detect the looseness of the connection, and the feasibility was successfully validated by laboratory experiments. However, other research literature related to this connection was focused on the failure behavior and design characteristics for improving the fatigue life of the connections [12,13]. For example, Van Wittenberghe [14,15,16] conducted a series of experimental and numerical studies to improve the fatigue life of pipeline couplings. He found that the fatigue life of these couplings can be improved by two major methods: one is to reduce the local stress concentrations by optimizing the geometry of the threads, and the other one is to optimize the global shape of the connection to obtain a more uniform load distribution. 

Recently, impedance-based technologies have been widely used as a nondestructive evaluation (NDT) technique in the field of structural health monitoring [17,18,19,20,21]. The basic concept of the impedance-based damage monitoring method is to use high-frequency vibrations to locally monitor the changes in structural mechanical impedance caused by the presence of damage, especially at the incipient stage [22]. The application of impedance signatures for damage detection was first proposed by Liang et al. [23] and followed by many other researchers for various damage detection problems [24,25,26,27,28,29,30]. For example, the impedance-based technique has been reported many times in the literature for application to bolt looseness detection [31,32,33], and Shao et al. [34] developed a piezoelectric impedance frequency-shift-based method to estimate the bolt preload for the detection of bolt looseness in engineering structures. The experimental results showed that the frequency shift of the measured admittance signature has a linear relationship with the preload on the bolt. Wandowski et al. [35] investigated the application of the electro-mechanical impedance (EMI) method to a metal structure (i.e., a part of the trailing edge of a wing) with bolted and riveted joints and demonstrated that the EMI method with correlation distance or chessboard distance seem to be a valuable local method for damage detection, even in changing temperature conditions, for geometrically more complicated structures. Huo et al. [36] combined the EMI technique with a “smart washer (SW)” fabricated by embedding a piezoceramic transducer into two flat metal rings to investigate the preload condition of a bolted connection. Liang et al. [37] and Fan et al. [38] conducted numerical and experimental investigations to monitor the looseness of pin-connected structures using the EMI technique, and the results showed that the changes in the measured impedance signatures consist of peak splitting and frequency shifts, which can be used to detect the looseness of pin-connected structures. For bolted connections in pipeline structures, Park et al. [39] experimentally investigated the feasibility of impedance-based health monitoring on civil pipeline structures connected by bolted joints, and data collected from the tests validated the capability of this technique for tracking and monitoring the integrity of a typical civil facility. Martowicz et al. [40] discussed the effectiveness of an EMI-based structural health monitoring (SHM) system for bolt loosening detection in a pipeline section with use of both point and transfer frequency-response-function (FRF) measurement, and alarm threshold levels based on transfer characteristics were proposed to allow for more variation and offer more reliable inferences on the condition of the monitored system. Though EMI-based techniques have been reported to solve many structural-health-monitoring-related projects, use of this technique for the loosening monitoring of threaded pipeline structures has not been reported in the literature.

In this study, the EMI-based technique was first employed to monitor the health condition of threaded connections in pipeline structures. In addition to studying the effectiveness of the developed method, this research also conducted the following studies which have not previously been reported in the literature:

(1) Investigation of the relationship between the contact area of the screwed interface and the electrical impedance signature of the lead zirconate titanate (PZT) patch bonded on the pipe coupling.
(2) Numerical simulations of loosening monitoring for the threaded pipe connection using the developed EMI-based approach.
(3) Quantitative evaluation of the connection loosening severity based on both experimental and numerical results.

In this research, two pipe segments coupled with a threaded fitting were assembled in the laboratory, and a PZT patch was surface bonded onto the coupling part. One pipe segment was artificially rotated anticlockwise with different rotation circles to simulate different degrees of loosening severities. For each loosening condition, experimental investigation using the developed piezoceramic-based EMI technique was performed, and the corresponding electrical impedance signatures measured from the PZT patch were collected. Subsequently, the root-mean-square deviation (RMSD) method was employed to build a looseness index to quantitatively present the different loosening severities. In addition, numerical simulations were performed to further investigate the effectiveness of the developed approach. Finally, the experimental and numerical results were compared to each other, and the feasibility of the EMI-based technique for loosening monitoring of threaded pipe connection was discussed. 

## 2. Detection Principle

### 2.1. Threaded Pipe Connections

**Figure 1:**
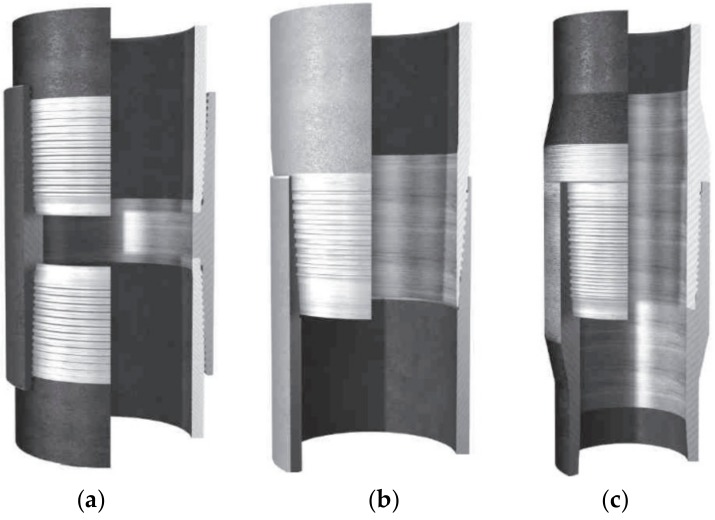
(**a**) Threaded and coupled; (**b**) integral flush; (**c**) integral upset connection [43].

A threaded pipe connection consists of a male and a female part, called the pin and box, respectively. Threaded connections can be divided into three main categories based on their method of manufacturing [41,42]. These three types are illustrated in Figure 1. The first type is called threaded and coupled (T&C), as shown in Figure 1a, which has male threads machined at both ends of the pipes and a separate coupling part is applied to assemble them together. This kind of connection is commonly applied in tubing, casing, and rise applications. The second type is called the integral type, since no separate coupling part is used for these connections, and the pipes have a male and female part of the connection at either end. In the case where the connection is fabricated in the pipe material without any local increase inside or outside the diameter, it is called a flush connection, as shown in Figure 1b; this is more commonly used in casing pipes. On the other hand, when the connection is fabricated in a part with a thicker wall than the rest of the pipe, it is called an upset connection, as shown in Figure 1c; this is used in tubing and drill pipes [16]. In this study, the first type of pipeline connection was selected and investigated to study the feasibility of the developed loosening monitoring method.

### 2.2. The Electro-Mechanical Impedance Technique

A PZT transducer possesses both actuating and sensing capabilities, and can therefore function as an actuator and a sensor, simultaneously [44,45,46,47,48]. For example, piezoceramic transducers acting as an actuator can produce a mechanical strain when subjected to an electrical field. Conversely, an electrical charge is produced when the piezoceramic transducer is mechanically stressed [49]. For the piezoceramic-based impedance technique, both the direct and converse characteristics of the piezoelectric effect were utilized to obtain the electrical impedance signatures of the piezoceramic transducers. 

Assuming that a fixed, alternating electric field is applied to a piezoceramic patch which is attached to a structure, a small deformation is produced in both the PZT patch and the attached structure. Considering that the frequency of the excitation is very high (usually higher than 30 kHz), only a very local area of the structure can be affected by the excitation. The dynamic response of that local area reflects back to the piezoceramic patch in the form of an electrical response. Thus, when a crack or damage occurs and changes the mechanical dynamic response of the host structure, i.e., a frequency phase shift or magnitude change in the mechanical dynamic response, the electrical response of the attached PZT patch can be utilized to detect this structural abnormality.

**Figure 2:**
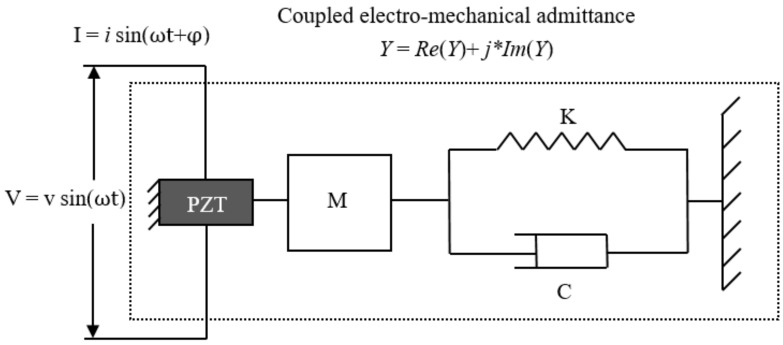
1D model representing a lead zirconate titanate (PZT)-driven dynamic structural system.

An electro-mechanical model which quantitatively describes the process is presented in Figure 2. Assuming that a surface-bonded PZT patch is attached to one end of the host structure represented by a single degree-of-freedom (DOF) system (i.e., a spring–mass–damper system), the other end of the PZT patch is fixed. The PZT patch considered to be a thin bar which vibrates axially when an alternating voltage is applied. Liang et al. [23] demonstrated that the electric admittance *Y*(ω) (which is an inverse of the electrical impedance *Z*(ω)) of the PZT actuator is a combined function of the mechanical impedance of the PZT actuator *Z_a_*(ω) and that of the host structure *Z*_S_(ω), as shown in the following equation:

(1) $$Y\left(\omega \right)=\frac{I}{V}=i\omega a\left({\bar{\varepsilon }}_{33}^{T}-\frac{Z_{S}(\omega )}{Z_{S}(\omega )+Z_{a}(\omega )}d_{3x}^{2}{\hat{Y}}_{xx}^{E}\right)$$

where *V* and *I* denote the input voltage and the output current of the PZT patch, respectively; *a* is the geometry constant of the PZT patch; *d_3x_* is the piezoelectric coupling constant in the arbitrary *x* direction at zero stress; ${\hat{Y}}_{xx}^{E}$ denotes the complex Young’s modulus of the PZT with zero electric field; and ${\bar{\varepsilon }}_{33}^{T}$ is the dielectric constant at zero stress. 

It is well known that damage causes the structural physical properties to change, including the mass, stiffness, and damping ratio, which directly affects the mechanical properties of the host structure. Assuming that the mechanical property of the surface-bonded PZT remains constant over the period of structural health monitoring, Equation (1) indicates that changes in the electrical impedance of the PZT patch can only be related to the mechanical impedance of the host structure. Therefore, any change in the electrical impedance signature of the PZT is considered an indication of a change in the structural integrity. 

Compared to global-vibration-based and other damage detection methods, the electro-mechanical-impedance-based technique has many advantages in structural health monitoring. The very high frequency (usually greater than 30 kHz) and low excitation force (less than 1 V) require power consumption in the range of microwatts. In addition, the small wavelengths due to the high frequency of the excitation provide the impedance-based method with an ability to detect slight changes in structural integrity and, in some cases, detect incipient damage. 

### 2.3. Loosening Monitoring for a Threaded Pipe Connection Using the EMI Technique

**Figure 3:**
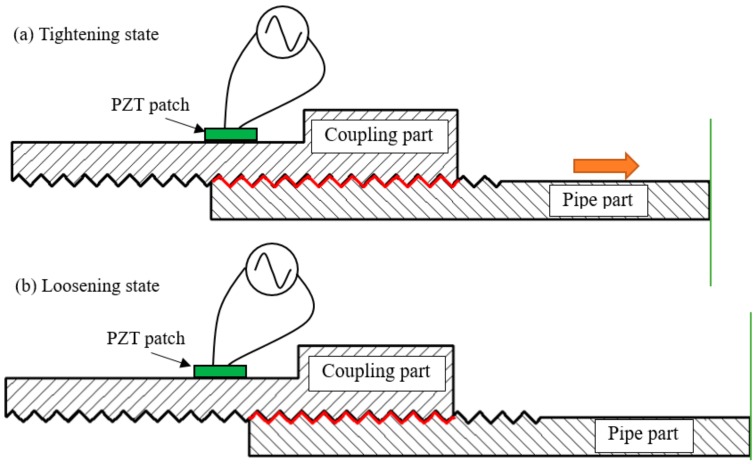
The changes in the contact area for the connection interface when looseness occurs.

For a threaded pipe connection, looseness always occurs with a decrease of the contact area of the thread interface between the coupling and pipes, as shown in Figure 3. Due to the change in the contact area, the variation of the structural mechanical properties, including the structural stiffness and damping ratio, will directly influence the mechanical impedance of the host structure. According to the PZT-driven impedance model introduced in Section 2.2, when a PZT patch is surface bonded onto the coupling of the threaded pipe connection, the PZT electrical impedance signature is coupled with the mechanical impedance of the host structure. Therefore, the looseness of the threaded pipe connection can be determined by monitoring the variation of the electrical impedance signatures of the PZT patch.

In order to quantify the changes in the impedance signatures of the PZT patch, statistical techniques such as the root-mean-square deviation (RMSD) [50,51,52] algorithm were employed in this study, as shown in Equation (2):

(2) $$RMSD=\sqrt{\frac{\sum_{i=1}^{N}{\left(Z_{i}^{1}-Z_{i}^{0}\right)}^{2}}{\sum_{i=1}^{N}{\left(Z_{i}^{0}\right)}^{2}}}$$

where $Z_{i}^{0}$ is the baseline impedance signature of the PZT patch, $Z_{i}^{1}$ is the corresponding post-damage value at the *i*th measurement point, and *N* is the number of measurement points. 

The RMSD index as an effective statistical algorithm was calculated and applied based on frequency-by-frequency comparison. Generally, a larger difference between the baseline signature and the subsequent test signatures results in a bigger value of the RMSD. In the area of structural health monitoring, RMSD-based damage index values denote changes in structural physical properties which may be caused by variation in geometrical and boundary conditions, environmental properties, and the presence of structural damage. For a damage detection method, larger RMSD values detected by a PZT patch may indicate a more serious change in the structural integrity.

In this study, the RMSD algorithm was employed to build a looseness index from the measured impedance signatures to monitor the looseness of pipeline fittings. For threaded pipe connections, looseness induces a decrease in the contact area of the thread interface and changes the electrical impedance signatures of the PZT patch surface bonded onto the coupling. In addition, with increasing loosening severity, the change in the measured impedance signature becomes greater and greater compared with that under the healthy condition, and finally induces a monotonic increase in the RMSD-based looseness index. Therefore, the looseness of the pipe connection can be identified and evaluated using the developed EMI-based technique by analyzing the changes in the RMSD-based looseness index. 

## 3. Experimental Investigation

### 3.1. Experimental Setup and Procedure

**Figure 4:**
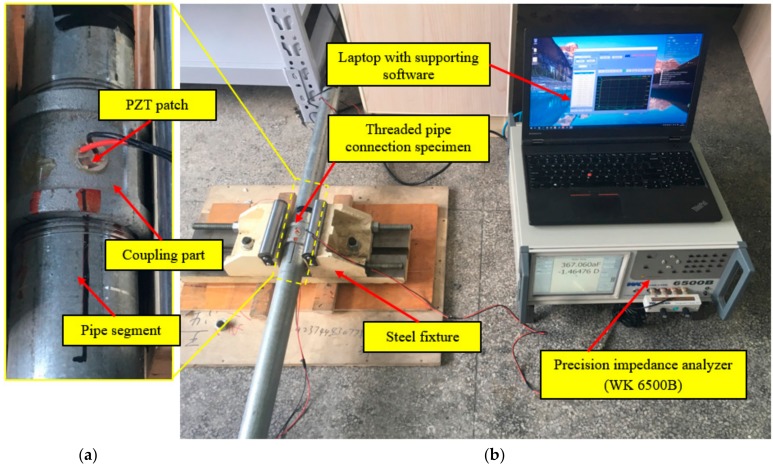
Experimental setup: (**a**) the test specimen and the PZT patch; (**b**) overall experimental setup.

Two pipe segments coupled with a threaded fitting were assembled in the laboratory to simulate the threaded pipe connection, as shown in Figure 4. The test specimen was fixed by a steel fixture. A PZT patch was mounted on the surface of the coupling part. The geometries and material parameters of the test specimen and PZT patch are shown in Table 1.

The experimental setup consists of the test specimen, a precision impedance analyzer (WK6500B, Wayne Kerr), and a laptop, as shown in Figure 4b. The PZT patch was surface bonded onto the coupling part of the specimen and then connected to the impedance analyzer. The impedance signatures were extracted as a function of the excitation frequency and the laptop was used to control the impedance analyzer.

**Figure 5:**
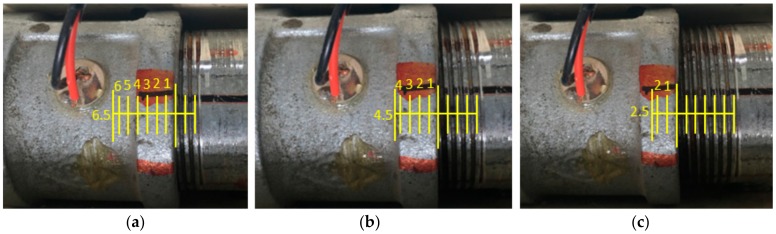
Several loosening severities for (**a**) 6.5 circles; (**b**) 4.5 circles; and (**c**) 2.5 circles.

In this study, the relative rotation circle between the coupling and the pipe segment was introduced as a monitoring variable to indicate the changes in the contact area of the threaded connection when looseness occurred and developed. Prior to the experiment, it was found that the threaded connection of the test specimen would get very tight after the contact region was greater than 6 circles by rotation of the pipe segment. Therefore, the initial tightening state (health baseline) for the connection in the experiment was fixed at 6.5 circles, then five different loosening severities were introduced by rotating the pipe segment with rotation circles of 5.5 circles, 4.5 circles, 3.5 circles, 2.5 circles, and 1.5 circles. Figure 5 gives three photos to demonstrate the different loosening conditions of the threaded pipe connection during the test. Here, the rotation circle directly determined the contact area of the thread interface between the coupling and pipe; for example, 6.5 circles means there are 6.5 circles of contact area for the thread interface, as shown in Figure 5a. Thus, a total of six working statuses including the initial healthy condition were implemented and investigated in this study. 

### 3.2. Experimental Results and Analysis

**Figure 6:**
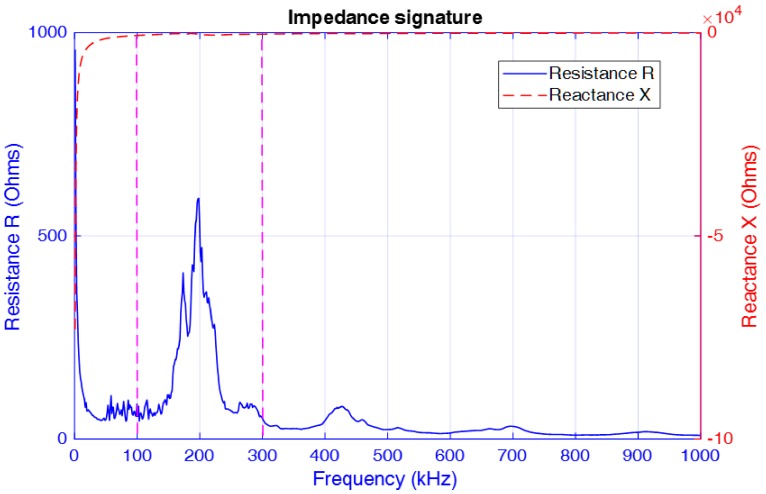
Electric impedance signature acquired from the PZT patch (1 kHz–1 MHz).

At the beginning of the experiment, an EMI-based broadband frequency sweep test with frequency variations from 1 kHz to 1 MHz was conducted for the specimen under the initial tightening (healthy baseline) condition, and the measured electrical impedance signature is presented in Figure 6. From the figure, it is clear that the real part of the electrical impedance signature (i.e., the resistance R) exhibits a sharp peak over the wide frequency range of excitation. This is consistent with the verified results in the literature [53] that the resistance signature is more sensitive to changes in the structural mechanical properties compared with the imaginary part of the impedance (i.e., the reactance X). This is due to the fact that the imaginary part of the impedance is dominated by the capacitive response of the PZT sensor and thus is less sensitive to changes in the mechanical properties of the structure. Therefore, the frequency range from 100 kHz to 300 kHz (i.e., the range between the two magenta dotted lines in the figure) was selected as the excitation frequency band for obtaining the electrical impedance of the PZT patch under the seven different loosening severities defined in the previous paragraph. These impedance signatures consist of 801 data points within the defined frequency range.

**Figure 7:**
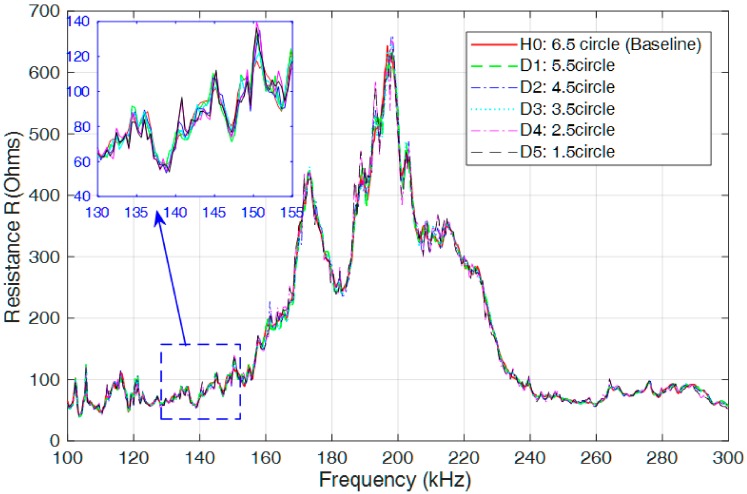
Measured electrical impedance signatures of the PZT patch (100 kHz–300 kHz).

In the experiment, a frequency-swept excitation signal with selected frequency band of 100 kHz to 300 kHz and voltage amplitude of 1 V was first generated and submitted by the precision impedance analyzer, and then transmitted to the surface-bonded PZT patch on the coupling part of the specimen. Under the excitation, a corresponding elastic wave was generated by the PZT patch and then propagated through the contact interface of the connection, interrogating the degree of looseness of the connecter structure, and providing information on the actual operating conditions of the threaded connection. Finally, the response signal with the structural health information was obtained by the PZT patch. Due to the piezoelectric effect, the impedance signature of the PZT patch was acquired and transmitted to the impedance analyzer. Thus, the looseness in the connection can be detected and monitored by comparing the measured impedance signatures with the one under the initial tightening (healthy) condition. Figure 7 presents the real part of the measured impedance signatures under the conditions of loosening severities from 6.5 circles (baseline) to 1.5 circles with intervals of 1 circle. A rectangular area in the figure was selected to zoom in to more clearly present the difference between the impedance signatures. 

**Figure 8:**
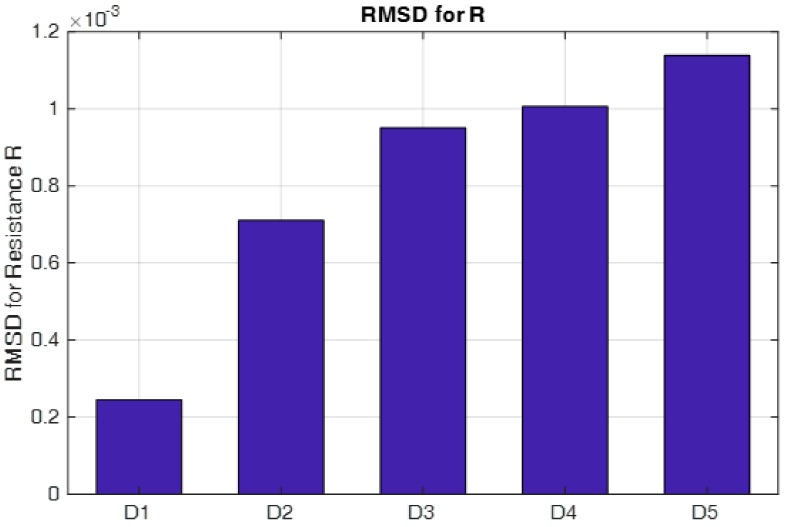
The calculated root-mean-square distance (RMSD)-based looseness index under different loosening severities.

From the figure, it is observed that there is a significate difference for the real part of the impedance signatures under different loosening severities. The root-mean-square distance (RMSD) algorithm was employed to build a looseness index to quantitatively distinguish the difference, and the final identification result is presented in Figure 8. In the figure, the RMSD-based looseness index increases with increasing loosening severity of the connection, especially at the initial or slight loosening conditions. This phenomenon can be explained by the characteristic of the high-frequency excitation, which is more sensitive to slight changes in structural integrity and, in some cases, can detect incipient damage. Based on the above analysis, it can be concluded that the developed piezoceramic-based EMI loosening monitoring method has the ability to detect the seal condition of the threaded pipe connection and to evaluate its loosening severity, especially in the case of slight change and incipient looseness in the connection.

**Figure 9:**
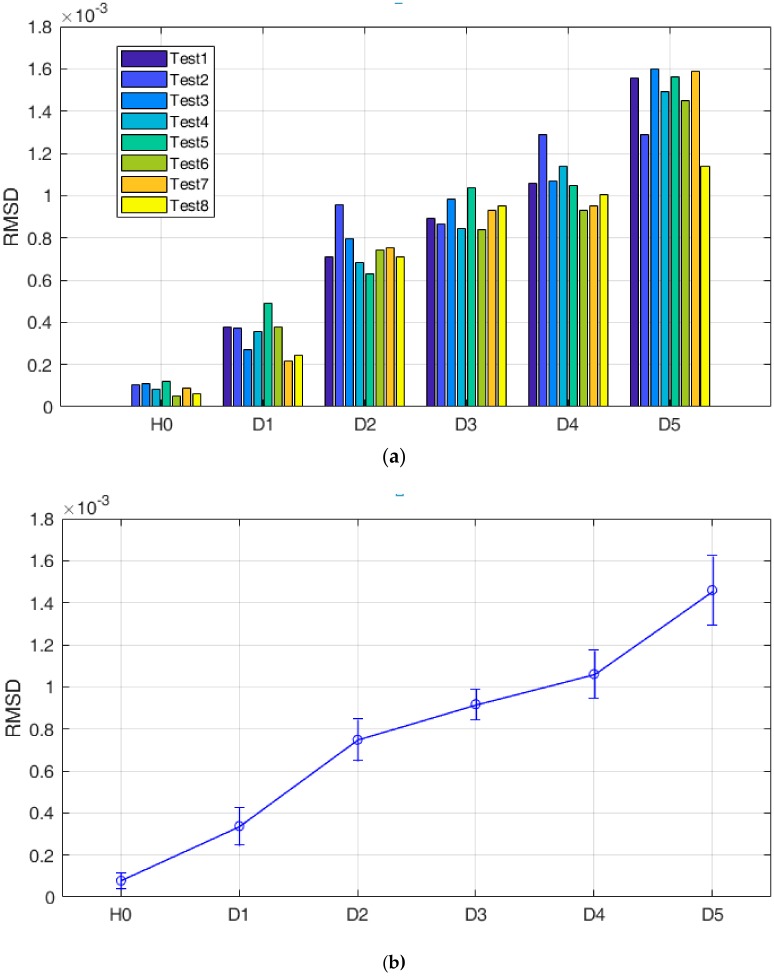
The eight repeated test results: (**a**) histogram; (**b**) error chart.

Repeatability of the proposed method and consistency of the test results are important for practical applications. Therefore, repeated tests, eight repetitions in this research, were investigated. The eight repeated tests were conducted in the same laboratory environmental conditions. Therefore, the environmental factors (such as temperature, moisture, pressure, etc.) were considered constant and did not affect the test results. A discussion of the influence of the environmental factors can be found in Section 4.3. Each time, the pipe segment was rotated from the tightest condition (6.5 circles, baseline, H0) to the loosest condition (1.5 circles, D5), and the proposed EMI-based loosening monitoring method was performed. The final eight repeated test results are shown in Figure 9 and Table 2, in which the mean values and standard deviations of the RMSD-based looseness indices under each loosening severity were calculated. On the other hand, considering the fact that the baseline impedance signatures play an important role in the procedure of RMSD-based index calculation, the stability and consistency of these baseline impedance signatures in the eight repeated tests were also investigated. 

From Figure 9a, it is observed that all the RMSD values in the eight repeated tests share a similar change trend, i.e., the proposed RMSD-based looseness index increased with the development of looseness when the pipe segment was rotated from the tightest condition (6.5 circles, H0) to the loosest condition (1.5 circles, D5). These results further validate the effectiveness of the proposed EMI-based loosening monitoring method in the study. At the same time, the mean values and standard deviations of these looseness indices under different severities were also calculated, as shown in Figure 9b and Table 2. From them, it is clear that the measured baseline impedances of these eight repeated tests present a good consistency (i.e., the lowest standard deviation) because of the tightest contact interface, which is beneficial to the following RMSD index calculation. However, as looseness develops, the standard deviation becomes larger, which indicates more serious dispersion for the detection results. It is predicted that the looseness development induces an unstable coupling connection and the interface contact area changes more randomly, which directly induces more serious dispersion for the RMSD-based detection results. In summary, all the results of the eight repeated tests demonstrate that the proposed EMI-based loosening monitoring method has relatively good repeatability and consistency and has potential to be used in the practical applications. 

## 4. Numerical Simulation

### 4.1. Threaded Connection Model

In order to further verify the effectiveness of the developed piezoceramic-based EMI technique for monitoring the looseness of threaded pipe connections, a numerical simulation of the connection structure based on a finite element model (FEM) was carried out using the commercial software ABAQUS, and the corresponding loosening monitoring investigation was performed. 

**Figure 10:**
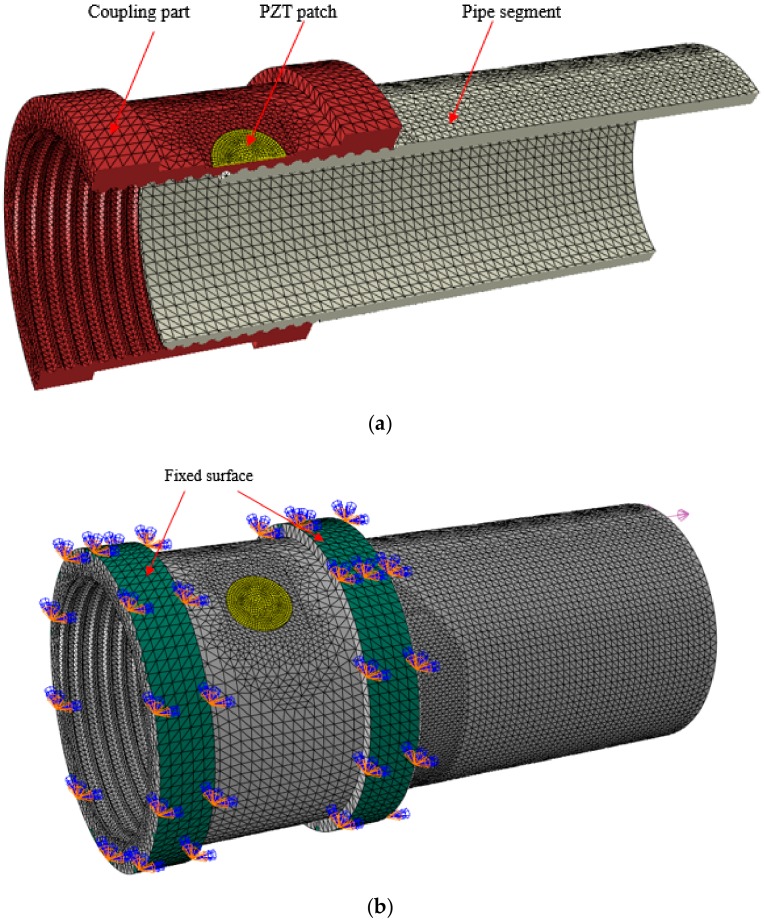
Geometry of the model: (**a**) the cross section drawn; (**b**) the boundary of the model.

As shown in Figure 10a, a coupling and a pipe segment were first established and assembled together as the test specimen, and a PZT patch was also established and then bonded on the outside surface of the coupling. All the geometric details and material properties of the connection model and PZT patch are the same as those in the previous experimental investigation, as shown in Table 1. For the boundary conditions of the model, two surfaces of the coupling were fixed in the investigation, as shown in Figure 10b.

After the finite element model was established, the electrical impedance of surface-bonded PZT transducers in the connection could be found with the help of ABAQUS software using the analysis command “Steady-stats dynamics, Direct”. On the other hand, the screw threads as the contact interface between the coupling and the pipe segment play a very important role in the EMI-based analysis procedure, so the mesh size of this contact surface was preset with the approximate global size of 2 mm and the minimum size controlled by a fraction of the global size of 0.1, which was smaller than the other parts of the model with the global size of 2 mm. The approximate global mesh size of the PZT patch in the numerical model was 0.5 mm, which was the thickness of the real PZT patch. All the mesh sizes of the numerical model were determined by considering the ultrasonic wave velocity in steel [54,55].

**Figure 11:**
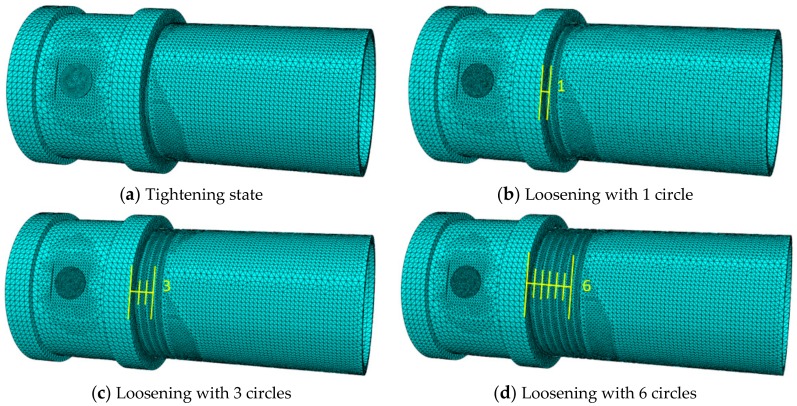
The simulated loosening conditions for the threaded pipe connection.

**Figure 12:**
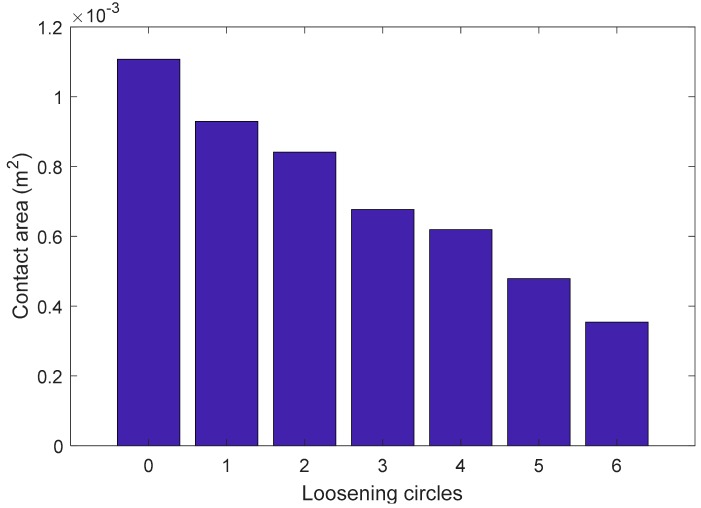
The relationship between the loosening rotation circles and the contact area.

In the simulation investigation, the coupling and the pipe segment were well engaged by the tooth bite, as shown in Figure 10a, and the loosening severities were introduced by rotating the pipe segment along the loosening direction with different rotation circles from 0 to 6 circles, as shown in Figure 11. Here, when the coupling was well tightened and no looseness occurred, the rotation circle of looseness was zero, as shown in Figure 11a, which was regarded as the healthy baseline condition in the following analysis. From the figure, it is clear that the contact area of the thread interface between the coupling and the pipe segment decreases with the development of loosening severity. In the simulation procedure, the contact area of the connection with different rotation circles was calculated and collected, as shown in Table 3 and Figure 12. 

### 4.2. Simulation Results and Analysis

As described in Section 2, when looseness occurs, the changes in the contact area of the thread interface of the connection have a direct influence on the structural physical parameters, such as the structural stiffness and damping ratio, which determine the mechanical impedance of the host structure. According to the theory of the EMI technique, variation in the mechanical impedance of the host structure can be detected from changes in the electrical impedance signatures of the surface-bonded PZT patch on the host structure. 

**Figure 13:**
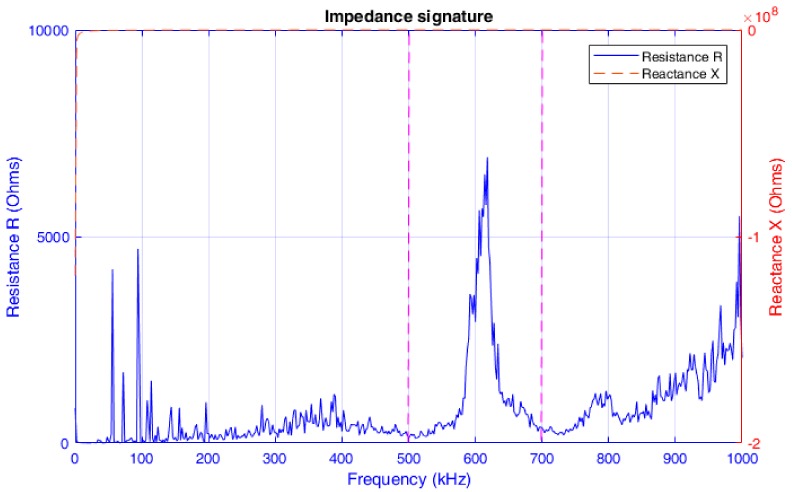
Electric impedance signature acquired from the PZT patch (1 kHz–1 MHz).

Prior to the simulation investigation, a swept-frequency excitation with a voltage magnitude of 1 V and a frequency band of 100 Hz to 1 MHz was applied to the PZT patch, and the corresponding impedance signatures were then acquired and analyzed, as shown in Figure 13. Thus, the sensitive frequency range of 500 kHz to 700 kHz was determined based on the impedance analysis result. 

**Figure 14:**
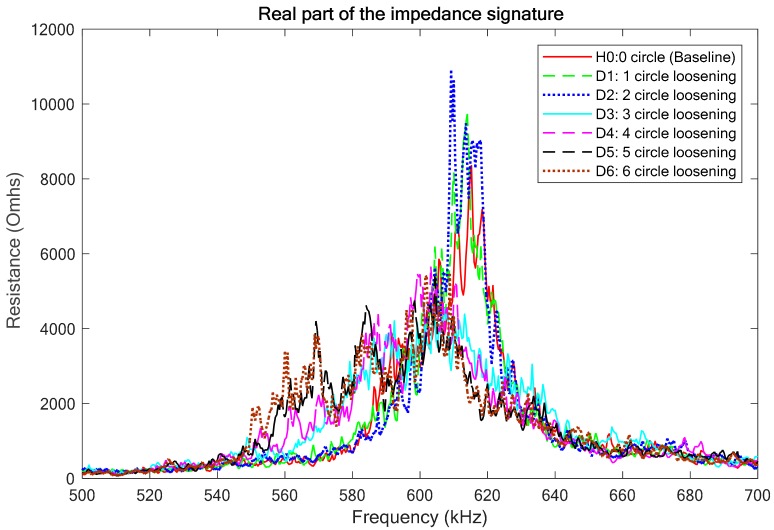
The real part of the impedance signatures for the threaded pipe connection model with different loosening circles (500 kHz–700 kHz).

**Figure 15:**
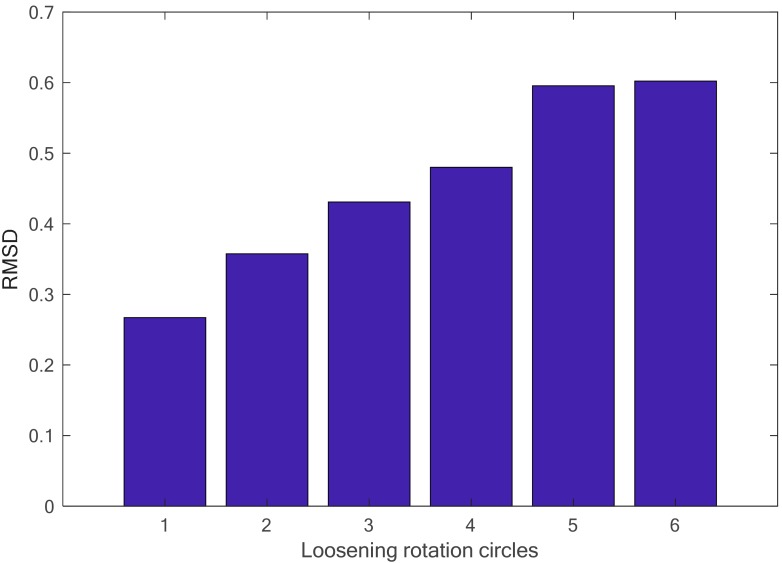
The identification result using the RMSD-based looseness index (500 kHz–700 kHz).

In the investigation, for each loosening severity with different rotation circles, the EMI-based swept-frequency excitation for the selected frequency band of 500 kHz to 700 kHz was generated and applied to the PZT patch, while the response electrical impedance signatures of the PZT patch were acquired and collected, as shown in Figure 14. The difference in the impedance signatures was distinguished using the RMSD algorithm, and the final identification result with the RMSD-based looseness index is presented in Figure 15. It is obvious that the looseness index increases with increasing number of loosening rotation circles, especially in the case of slight looseness. The simulation result has a similar change trend with that of the experimental investigation; thus, an identical conclusion can be drawn that the developed piezoceramic-based EMI method has the capacity to detect looseness and to evaluate its severity to some degree, especially in the case of the slight condition for the threaded connection. 

**Figure 16:**
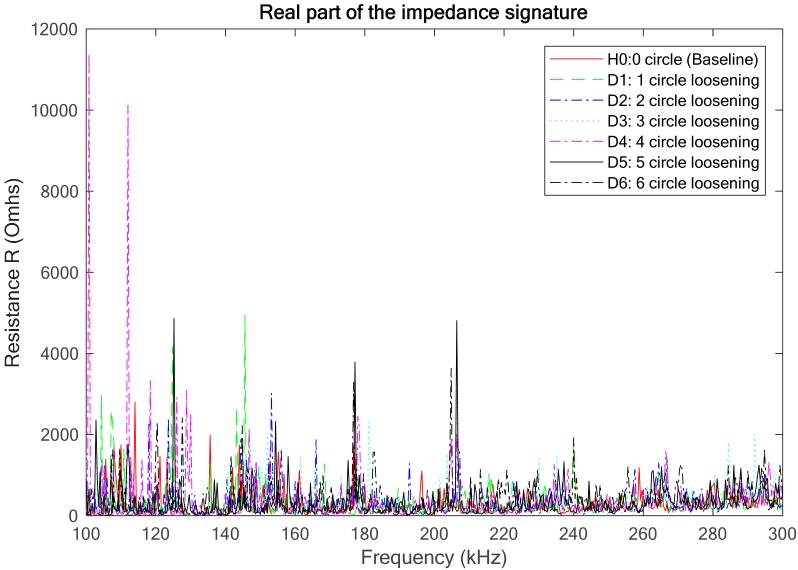
The real part of the impedance signatures for the threaded pipe connection model with different loosening circles (100 kHz–300 kHz).

**Figure 17:**
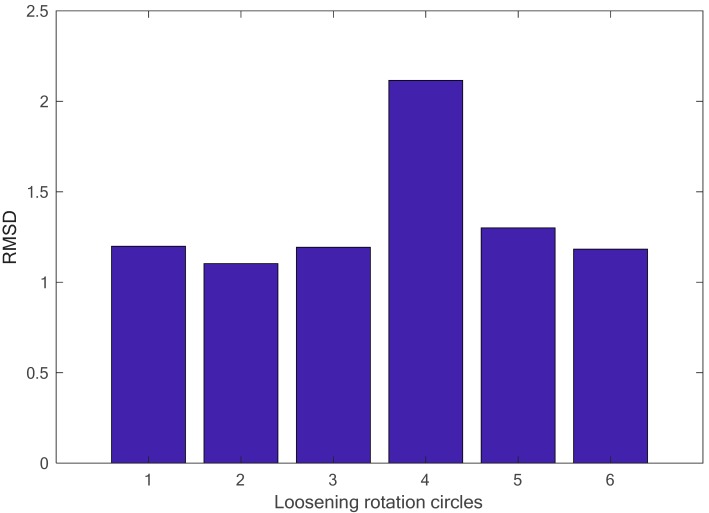
The identification result using the RMSD-based looseness index (100 kHz–300 kHz).

In the study, the feasibility of the proposed EMI-based method was investigated by experimental tests and numerical simulation. However, the impedance sensitive frequency band in the numerical simulation was selected as the range of 500 kHz to 700 kHz, which was different from that in the experimental investigation (100 kHz to 300 kHz) due to some reasons. In order to further compare the simulation results with the experimental test in the same frequency band, the impedance signatures of the numerical model for the impedance frequency band of 100 kHz to 300 kHz were also acquired and analyzed, as shown in Figure 16. Then, the RMSD-based looseness index under this condition was calculated, as shown in Figure 17. From the figures, it is observed that neither the impedance signatures nor the RMSD-based looseness index analyzed under the impedance frequency band of 100 kHz to 300 kHz present a significant change in trend compared to the above results presented in Figure 14 and Figure 15. This phenomenon may be caused by the differences between the numerical model and the experimental specimen, and a detailed discussion of these differences appears in Section 4.3. Based on the above analysis, a conclusion can be drawn that selecting an appreciate impedance sensitive frequency band is very necessary and important for EMI-based damage detection techniques and will seriously affect the final identification results. 

### 4.3. Discussion

In this research, a feasibility study on loosening monitoring of threaded pipe connections using the EMI technique was conducted. The results from both the experiment and simulations show good agreement that the RMSD value of the looseness index has a positive correlation with the development of the loosening severity. However, the index values for the same looseness condition from experimental and numerical results differ. There are some possible reasons for this phenomenon. On the one hand, the boundary conditions for the experimental specimen and the numerical model are not exactly the same. On the other hand, the geometrical parameters for the pipe segment (length, wall thickness, etc.) and the screw thread (thread pitch, taper, and chamfer, etc.) are all different. All these differences directly influence the final EMI-based identification results, including the selected impedance-sensitive frequency range and the measured impedance signatures, etc., as shown in Figure 7, Figure 8, Figure 13 and Figure 14. 

Besides this, it should be noted that the Lamb wave propagation and the final detection results of the developed piezoceramic-based EMI method will also be influenced by some other factors, including thermal stress, moisture, environmental pressure, sensor location, and structural materials, etc. For example, several researchers have experimentally verified that changing temperature will induce differences in EMI-based detection results, including magnitude changes or frequency shifts for the measured impedance signatures [56,57]. In the current state, due to space limitations, the study is focused on the feasibility verification of using a piezoceramic-based EMI technique to detect the looseness of a threaded pipe connection. Implementation of the developed method to loosening monitoring of a real threaded pipe connection remains a future topic of study. 

## 5. Conclusions

In this study, a piezoceramic-based active sensing method combined with an EMI technique was developed to detect the looseness of a threaded pipe connection. A connection test specimen assembled from a coupling and two pipe segments was prepared and experimentally investigated in the laboratory; meanwhile, a finite element model of this threaded connection model was established and analyzed. One PZT patch was surface bonded onto the coupling to acquire the electrical impedance signatures. Finally, the results from both the experimental investigation and the numerical simulation have good consistency with each other, i.e., the RMSD-based looseness index increases with the development of the loosening severity. Therefore, it has been demonstrated that the developed piezoceramic-based EMI technique has great potential for determining early looseness of threaded connections, as well as for monitoring the health status of the pipeline fitting during its service life. 

## Figures and Tables

**Table 1:** The parameters of the specimen and the PZT patch.

Components	Parameters	Values	Unit
Steel specimen	Diameter (pipe segment)	∅48 (∅42) ^1^	mm
	Diameter (coupling part)	∅60 (∅45) ^1^	mm
	Density	7900	Kg/m^3^
	Young’s modulus	206	Gpa
	Poisson’s ratio	0.3	—
	Static friction coefficient (steel–steel)	0.15	—
PZT-5H	Dimension	∅12× 0.5	mm
	Density	7800	Kg/m^3^
	Young’s modulus	46	Gpa
	Poisson’s ratio	0.3	—
	Structural damping	3 × 10^−9^	—
	Dielectric loss factor	0.02	—
	Mechanical loss factor	0.001	—
	Piezoelectric strain coefficients *d*_31_, *d*_32_/*d*_33_/*d*_24_, *d*_15_	−2.10/5.00/5.80	10^−10^ m/V or 10^−10^ C/N
	Electric permittivity $\varepsilon_{11}^{T}$, $\varepsilon_{22}^{T}$/$\varepsilon_{33}^{T}$	1.75/2.12	10^−8^ F/m

^1^ the value in parentheses donates the inner diameter.

**Table 2:** The means and standard deviations for repeated tests.

Status	Mean	Std.
H0	7.79 × 10^−5^	3.90 × 10^−5^
D1	3.37 × 10^−4^	8.86 × 10^−^^5^
D2	7.48 × 10^−4^	9.86 × 10^−^^5^
D3	9.17 × 10^−4^	7.15 × 10^−^^5^
D4	1.06 × 10^−3^	1.14 × 10^−^^4^
D5	1.50 × 10^−3^	1.64 × 10^−^^4^

**Table 3:** The contact area of the thread interface with the increase of loosening severity.

Loosening Rotation Circles	0	1	2	3	4	5	6
Contact area (×10^−4^ m^2^)	11	9.29	8.41	6.77	6.19	4.79	3.54
